# Rare Case of Anterior Sacral Meningocele in a 30‐Year‐Old Male: Surgical Excision Using the Posterior Sagittal Approach

**DOI:** 10.1002/ccr3.71990

**Published:** 2026-02-09

**Authors:** Ayesha Farooq, Zaryab Bacha, Umaima Cheema, Maryum Farooq, Abdullah Afridi, Fathimathul Henna, Muhammad Abdullah Ali, Kamil Ahmad Kamil

**Affiliations:** ^1^ Khyber Medical College Peshawar Pakistan; ^2^ King Edward Medical University Lahore Pakistan; ^3^ Ayub Medical College Abbottabad Pakistan; ^4^ Dubai Medical College for Girls Dubai UAE; ^5^ Department of Internal Medicine Mirwais Regional Hospital Kandahar Afghanistan

**Keywords:** anterior sacral meningocele, case report, posterior sagittal approach, presacral mass, spinal dysraphism, urinary retention

## Abstract

Anterior sacral meningocele (ASM) is an uncommon congenital spinal abnormality in which the meningeal sac herniates through an abnormality in the anterior sacrum into the presacral space. It is more observed in females and usually asymptomatic; large ASMs may cause pressure effects on nearby pelvic structures. A 30‐year‐old male presented with prolonged constipation and recurrent episodes of urinary retention, which developed to complete urinary obstruction. Ultrasound demonstrated a large presacral cystic mass, and MRI confirmed the diagnosis of an ASM. Pelvic x‐ray revealed the classic Scimitar Sign. Neurological examination showed no abnormalities. The patient was subjected to surgical excision through the posterior sagittal technique. Intraoperative steps consisted of laminectomy, durotomy, sac decompression, and fascial graft repairing. After the procedure, the patient experienced a smooth recovery with notable improvement in bowel and urinary symptoms. ASM is an uncommon etiology of pelvic mass and neurogenic bladder symptoms occurring in adults. This case underscores the diagnostic importance of MRI and pelvic X‐ray, and also supports the posterior sagittal approach as an effective, low‐complication surgical approach. ASM should be taken as a differential diagnosis for adult patients having symptoms of chronic constipation and urinary retention. Prompt imaging and surgical treatment can notably improve outcomes.

## Key Clinical Message

Anterior sacral meningocele is a rare cause of chronic constipation and urinary retention in adults. MRI is essential for diagnosis, with the scimitar sign as a helpful radiologic clue. The posterior sagittal approach provides safe and effective surgical management with excellent outcomes.

## 1 Introduction

Anterior sacral meningocele (ASM) is a rare spinal dysraphism in which the meninges herniate into the retroperitoneal and presacral spaces through a defect in the anterior sacral wall [1]. ASM may be asymptomatic, with symptoms emerging later in life, or it may present early with clinical signs resulting from compression of adjacent pelvic viscera. Meningoceles are commonly located at the thoracic and lumbosacral spinal levels, predominantly posterior. They account for approximately 10% of all cases of spina bifida [2]. This case highlights the necessity of evaluating ASM in patients presenting with a lower abdominal mass and associated urinary symptoms. Here we report a rare case of ASM in a 30‐year‐old male excised using the posterior sagittal approach.

## 2 Case Presentation

A 30‐year‐old male presented with a long‐standing history of chronic constipation and intermittent urinary retention, persisting for several years. Despite these persistent symptoms, he had only sought occasional outpatient consultations and had never been hospitalized. No prior imaging had been performed, and he had not received any long‐term medications. His medical history was unremarkable, with no prior surgeries, or diagnosed neurological conditions.

In the weeks preceding his presentation, the patient experienced a progressive worsening of urinary symptoms, eventually leading to complete urinary retention, which required catheterization for relief. Concerned about the escalation of his condition, he sought medical evaluation. An abdominal ultrasound performed by a general surgeon revealed a large cystic presacral pelvic mass, estimated to measure approximately 1.5–2 L in volume. The patient was subsequently referred for further evaluation.

On physical examination, his abdomen appeared distended, with a firm, immobile, and non‐tender mass palpable up to the level of the umbilicus. The mass was dull to percussion, suggesting a deep pelvic origin. There were no signs of inflammation or overlying skin changes. Neurological examination of the lower limbs showed full motor strength (5/5), intact sensation across all dermatomes, including the perineal region, and normal deep tendon reflexes (2+). Anal sphincter tone was preserved.

**FIGURE 1:**
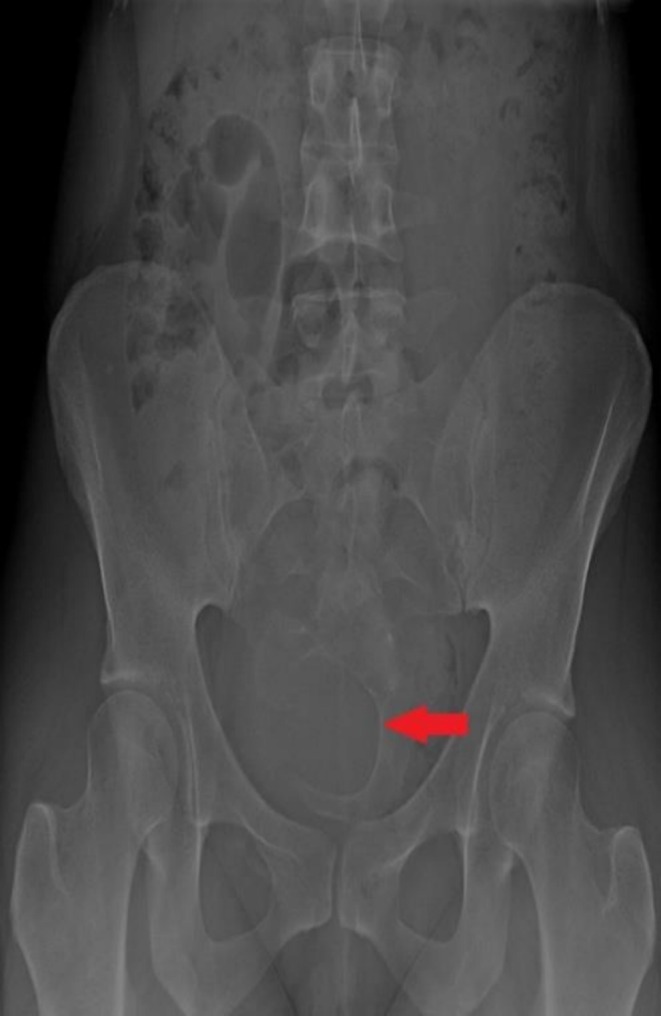
Plain pelvic x‐ray demonstrating the Scimitar sign characteristic of anterior sacral meningocele.

Baseline laboratory investigations, including complete blood count, were within normal limits, with no signs of infection or systemic inflammation. Urine routine examination showed no abnormalities, and renal function tests (blood urea and serum creatinine) were also normal, despite the patient's chronic urinary retention. Radiological evaluation included an MRI of the lumbosacral spine, which confirmed the diagnosis of ASM. Additionally, a plain pelvic x‐ray demonstrated the classic Scimitar Sign, supporting the diagnosis and guiding the need for surgical intervention (Figure 1).

## 3 Differential Diagnosis, Investigations, and Treatment

**FIGURE 2:**
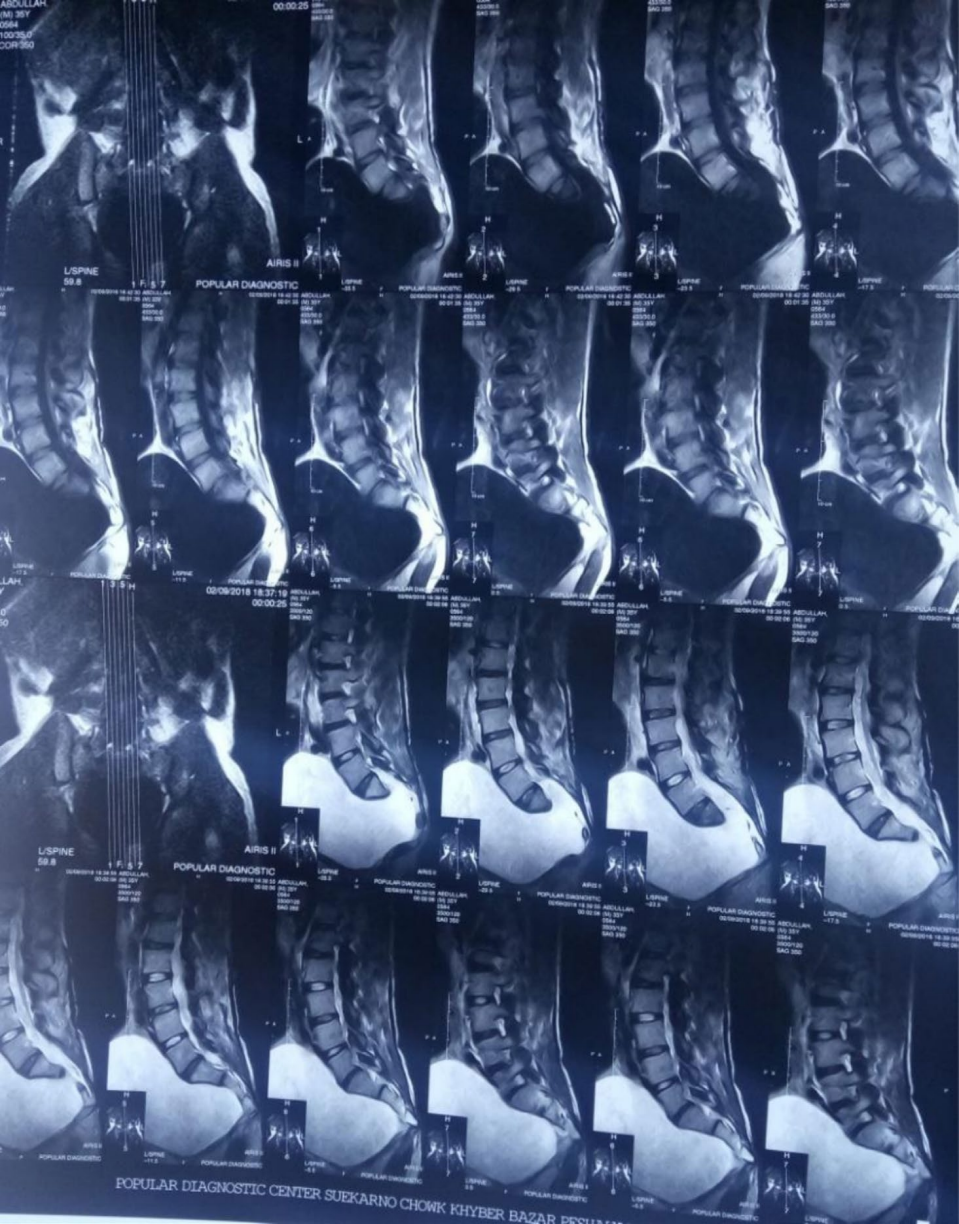
MRI Lumbosacral Spine showing a large anterior sacral meningocele sac occupying the pelvic cavity.

Radiological evaluation was initiated. A plain pelvic x‐ray revealed the classic *Scimitar Sign*, suggestive of a sacral anomaly. Subsequent MRI of the lumbosacral spine confirmed the diagnosis of ASM—a rare congenital defect characterized by herniation of the meningeal sac through a defect in the anterior sacrum (Figure 2).

**FIGURE 3:**
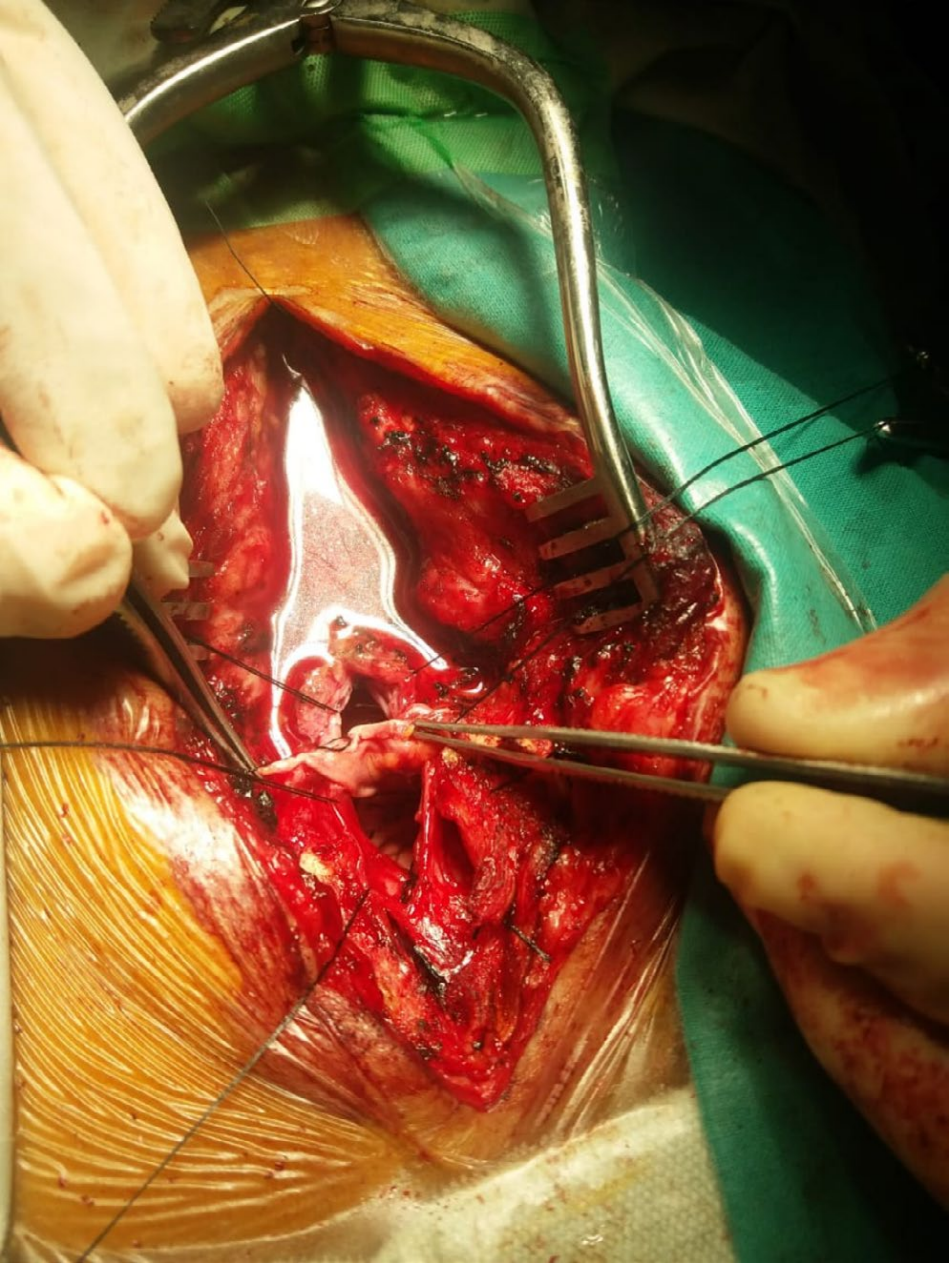
Intraoperative view of dural opening and CSF‐filled sac evacuation during posterior approach surgery.

Given the large size of the lesion and its impact on bowel and urinary function, surgical intervention was planned. The patient underwent resection and repair of the meningocele via a posterior midline approach. Under general anesthesia, a posterior midline incision was made, followed by laminectomy and durotomy to access the meningeal sac (Figure 3). The sac was carefully decompressed, and the dural defect was identified. Closure was achieved using a fascia graft with meticulous suturing to prevent recurrence. The procedure was completed without any intraoperative complications.

## 4 Conclusion and Results (Outcome and Follow‐Up)

**FIGURE 4:**
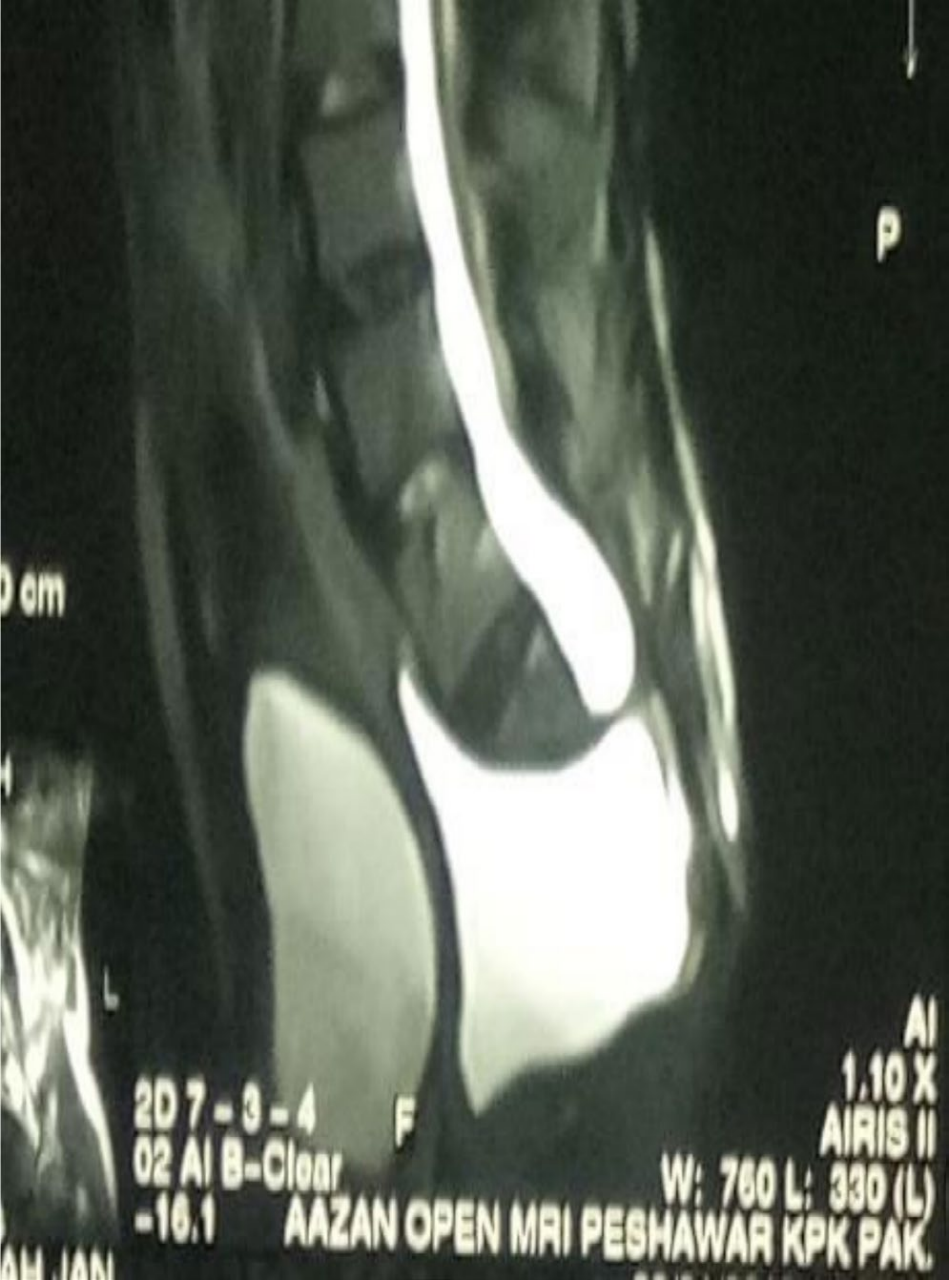
Postoperative image indicating decompression of the meningocele sac and recovery.

Postoperatively, the patient was managed with intravenous antibiotics, analgesics, and IV fluids. His recovery was smooth, with no immediate postoperative complications (Figure 4). The surgical wound remained clean and well‐healed. At the 2‐week follow‐up, stitches were removed, and there were no signs of postoperative infection. Subsequent follow‐ups at 1‐ to 3‐month intervals showed gradual improvement in bowel and urinary function. The previously noted swelling had completely subsided, and the patient reported a significant reduction in symptoms.

## 5 Discussion

Anterior sacral meningocele, abbreviated as ASM, is defined as a rare and clinically significant representation of “spinal dysraphism” in which the meningeal sac typically herniates into the presacral or retroperitoneal space via a defect in the anterior aspect of the sacrum [3, 4, 5]. They were first described in the 19th century and occur more commonly in females as compared to males [6]. ASMs are often linked or associated with various other conditions like sacral agenesis, Currarino syndrome, or some other caudal regression anomaly [3, 4]. Although this defect is congenital, ASM may remain asymptomatic until adulthood when it grows to a size to be able to compress the adjacent pelvic soft tissues and viscera [7, 8]. Our case is unique as it presents a male patient with late presentation of ASM, which is unique because of the significant volume of the lesion and successful management of the defect via the posterior sagittal approach.

ASMs account for only 5%–10% of all cases of meningoceles and are, thus, a small subset of spinal meningeal cysts [9]. Most of the cases having ASM present in the 20s or 30s which often get misdiagnosed as ovarian masses, pelvic cysts or even retroperitoneal tumors which, at one point or another, highlights the importance of awareness of rare cases to diagnose such conditions at appropriate time as well as proper management of such conditions and defects [10, 11]. Some common clinical features of ASM include lower abdominal discomfort, symptoms due to “mass” effect, urinary retention, constipation, perineal pain, or reproductive issues in females [8, 12, 13, 14]. In our case, the patient reports a prolonged history of constipation with intermittent urinary retention, which is a classic but often overlooked or neglected representative of symptoms in ASM, especially when it comes to male patients [15, 16].

One gold standard test for the diagnosis of ASM is radiological diagnosis, that is, MRI because of its precise soft tissue resolution and ability to perfectly visualize and delineate the connection between the cyst and the spinal cord [12, 17, 18]. In this case as well, the diagnosis was confirmed via MRI showing a large and cystic lesion lying at the presacral level communicating with the spinal thecal sac. The “Scimitar sign” was also positive on pelvic x‐ray, which is suggestive of sacral dysraphism as well [7, 19, 20]. Ultrasound and CT myelography might also be used, but carry a lower sensitivity as compared to MRI [21, 22].

Regarding the management of ASM, it depends upon the size of ASM, risk of complication and, most importantly, the symptoms. In patients with no symptoms of ASM or minimal symptoms, usually a conservative approach is preferred with observation [23, 24]. However, for symptomatic patients or patients having enlarging ASMs, surgery remains the final and definitive treatment. In our case, the decision was to subject the patient to an operation as there was progressive urinary retention with mass effect on pelvic organs. As far as the surgical approaches are concerned, they include transabdominal, posterior sacral, and combined approaches [13, 16, 18, 25, 26]. The approach used in this case was sagittal as it minimized the need for visceral dissection, provided direct access to the defect and led to effective excision and repair.

Regarding the surgical techniques employed in the correction of this defect, previous studies have discussed several merits and demerits regarding different techniques. Regarding the transabdominal approach, although it provides excellent access to large or multiloculated lesions, it also carries greater complications and risks [27]. In contrast, the posterior approach carries minimal complications and risk, prioritizing the overall safety, making it a better choice in this case for the surgical correction of ASM [16, 18, 28]. However, there is a need for long‐term monitoring to detect any possible recurrences or delayed cognition‐related and other issues.

One important point requiring special attention in this case is the delayed diagnosis. As ASMs carry various atypical symptoms, they may remain undiagnosed for several years or even a lifetime, which highlights the need of the hour to develop awareness among surgeons and clinicians regarding this rare case [29, 30]. Our study contributes directly to raising awareness along with a detailed investigation regarding this rare case, that is, ASM. Several other studies present in the literature also suggest including ASM in the differential diagnosis of urinary retention and chronic constipation, especially in young adults, where, usually, the conventional causes get excluded [31, 32]. In addition, this increased awareness regarding ASM with early detection via MRI can lead to better diagnosis, preventing misdiagnosis of the defect and can directly contribute to a reduction in complications like infections or infertility [33, 34].

When discussing global epidemiology, ASMs are found to largely remain unreported, with fewer than 300 cases documented in the literature, making this case a relevant one [12, 35]. Reports regarding this case from continents like Asia and low‐resource settings are particularly limited, which may be due to factors such as under‐recognition or various diagnostic constraints. This also highlights the importance of case reporting, as it contributes to enhanced recognition worldwide and improved early diagnosis. These points highlight the strengths of this case report as well. However, the report also contains certain limitations. Firstly, there was a lack of genetic evaluations to rule out possible familial or syndromic forms. Secondly, the postoperative follow‐up duration was quite limited. Thirdly, although the sagittal approach for surgery was employed in this case, it may not be suitable for all cases, making it universally inapplicable, especially in the presence of complex or recurrent ASMs. These limitations underscore the need for more prospective and multicenter studies, as well as the development of consensus guidelines regarding various surgical approaches.

Therefore, this case is highly significant as it contributes to the growing understanding of conditions like ASM in adults, particularly in diagnosing it in males, where it may remain unrecognized due to its atypical presentation. The study emphasizes the importance of MRI in diagnosing ASM and the effectiveness of the posterior sagittal approach for surgical correction. Thus, it not only encourages but also assists surgeons and clinicians by providing a detailed investigation and enhancing their awareness of this rare condition. Nonetheless, there is a need for improved interdisciplinary management, ongoing efforts for early diagnosis, and standardized surgical options to significantly enhance the outcomes related to this impactful yet rare case.

## Author Contributions

**Ayesha Farooq:** conceptualization, supervision, writing – review and editing. **Zaryab Bacha:** data curation, writing – original draft, writing – review and editing. **Umaima Cheema:** writing – original draft. **Maryum Farooq:** writing – original draft. **Abdullah Afridi:** data curation, writing – review and editing. **Fathimathul Henna:** writing – original draft. **Muhammad Abdullah Ali:** writing – review and editing. **Kamil Ahmad Kamil:** conceptualization, supervision, writing – review and editing.

## Funding

The authors have nothing to report.

## Consent

Written informed consent was obtained from the patient according to the journal guidelines.

## Conflicts of Interest

The authors declare no conflicts of interest.

## Data Availability

All data used in this study are cited within the manuscript.
